# Simulation and Optimization of the Antenna Designs for Glucose Biosensing FRET Mechanisms in Endoscopic Capsules

**DOI:** 10.3390/mi16060641

**Published:** 2025-05-28

**Authors:** Rajaa B. Naeem, Doğu Çağdaş Atilla

**Affiliations:** 1 Department of Electrical and Computer Engineering, Altinbas University, 34217 Istanbul, Türkiye; 2 Electrical-Electronics Engineering Department, Faculty of Engineering and Architecture, Altinbas University, 34217 Istanbul, Türkiye; cagdasatilla@gmail.com; 3 Data Analytics Department, Institute of Graduate Studies, Altinbas University, 34217 Istanbul, Türkiye

**Keywords:** FRET-based biosensing, glucose sensing, photodetector optimization, CST Studio Suite, SAR calculation, antenna design, endoscopic capsule, visible optical frequency, non-invasive glucose monitoring

## Abstract

An optimized design of photodetectors and antennas for Förster Resonance Energy Transfer (FRET)-based glucose biosensing in endoscopic capsules is presented. The compact antenna design is tailored for the visible optical frequencies (~526 THz) associated with FRET-based glucose monitoring and integrates structural flexibility to conform to the spatial constraints of endoscopic capsules, such as mechanical bending features. The antenna is embedded in a multimode medium artificial tissue simulating a glucose environment with several layers, providing efficient coupling to the FRET emission signal for glucose sensing. Stable S11 parameters and a maximum gain of 9 dBi are realized by statelier mesh settings, bend adaptation, and cautious SAR constraint handlers. Results of the Specific Absorption Rate (SAR) confirm the limited energy absorption within permissible bounds, confirming its application for biomedical purposes. These results affirm the feasibility of non-invasive glucose measurement in interstitial fluid in this configuration that can be operable through an endoscope with improved sensitivity and functionality.

## 1. Introduction

The monitoring of glucose levels is important in the management of diabetes and the diagnosis of other metabolic disorders [1]. However, current methods are mostly invasive, requiring blood samples. Although effective, these methods are uncomfortable, intrusive, and risk infection, thus necessitating the development of a non-invasive monitoring method [2]. An advance in sensor and biosensing technologies enables the development of non-invasive, patient-friendly glucose monitoring methods [3]. New fluorescent biosensors based on the use of quantum dots (QDs) have been discovered to have a more sensitive response for biological applications in comparison with other works. The proposed study is based on these findings, and the authors investigate the challenges that can be addressed based on the study by Bai et al. [4].

One such non-invasive approach is using Förster Resonant Energy Transfer-based biosensing. This method relies on the transfer of energy between two fluorescent molecules; the energy transfer can also be influenced by analytes, such as glucose [5]. This FRET biosensing method is sensitive and specific and can be used in wearable/non-removable monitoring devices, such as endoscopic capsules, which allow for easy access to glucose measurements [6].

Implementing FRET-based biosensing in an endoscopic capsule presents difficulties due to restricted space and the necessity of photodetection with precision and accuracy [7]. Optimizing the photodetector design for operation within the endoscopic capsule requires the development of an optimized photodetector model. Sensors that are both spatially suitable for use in endoscopic capsules and capable of detecting signals at low energy levels are required.

In the most optimal scenario, the design of FRET-based endoscopic biosensors would allow operation without a power supply over the entire measurement duration [8]. Such optimization requires the consideration of specialized wavelength requirements for FRET and implementation of materials suitable for space constraints, i.e., manufacturing the photodetector with space constraints in mind. Delivering an optimized model that meets all of these criteria requires extensive simulations.

This work is a simulation study performed using CST Studio Suite 2019. In order to create the photodetectors, it is necessary to simulate the electromagnetic interactions in the detector and produce an optimization of the antenna that would hold a positive dielectric field. This can involve adjustments of the antenna’s size, shape, or the materials from which it is produced. After that, the photodetectors are produced as a part of the antenna designs that affect the antenna operation to the best degree. The purpose of the present study is the optimization of the photodetector design for FRET-based glucose biosensing within endoscopic capsules in regard to signal gain and mechanical robustness under practical limitations.

This paper is structured to first provide a literature review: glucose monitoring devices, methods of FRET, and methods of photodetection simulation are discussed in Section 2. In Section 3, the developed design of FRET-based biosensors is introduced, together with the method of CST simulation. The simulation results and their interpretation are discussed in Section 4, while in Section 5, the results are consolidated and summarized with some implications for biosensor development.

## 2. Literature Review

Terahertz (THz) technology is known as a promising candidate for biomedical applications, especially sensors and diagnostic devices. Additionally, Malhotra and Singh [9] offer a review of THz antenna technology for imaging and sensing, as well as the opportunity for high-resolution imaging for human diagnostics. As explained by Pandey [10], the dual-substrate graphene-based THz antennas provide even more effective functional features of the THz system in high-frequency biomedical applications, thus opening up a way for the design of better-performing antennas. On the other hand, in recent years, the use of photoconductive antennas for biomedical imaging [11] illustrates that the ultra-broadband pulses generated are appropriate for the demanding frequencies in many diagnostic devices. While these works primarily focus on THz frequencies (0.1–10 THz), the present study extends the concept to the visible optical frequency range (~526 THz) to match the spectral characteristics of FRET-based biosensing.

Fluorescence resonance energy transfer (FRET)-based biosensors have also advanced to the point of real-time monitoring of glucose and other biomarkers for diagnostics. In [12], Singh et al. reviewed principles and applications of FRET-based biosensors, which can be potentially used for non-invasive diagnostics. Example applications are the creation of a FRET-based biosensor to detect glucose content in the fungal cultivations of Otten et al. [13]. However, in [13], the researchers show that such sensors can be used in the industrial and medical fields. Furthermore, Keller et al. [14] present structural analyses of genetically encoded FRET biosensors. Human molecular design and functional optimization of genetically encoded FRET biosensors.

While THz technology and FRET-based biosensors have both advanced considerably, the theoretical coupling of these has remained an open area. Zhang [15] developed a new type of THz time–domain spectroscopic endoscope-based single photoconductive antenna chip. When discussing their technique, they speculated it may provide the ability for in vivo application. Building on this concept, Danasegaran [16] employed a THz antenna based on a high-frequency photonic crystal and a design for biomedical spectroscopic applications. Moreover, Nagand Kaur [17] proposed a rectangular slit microstrip patch antenna at 1.752 THz, which was tested and verified to apply in biosensing systems.

The authors of [18] summarize important computational and simulation studies that have critically contributed to the realization of THz antenna designs. Jamshed et al. [18] introduced a computational model for THz antennas that is specifically tailored to some of the challenges unique to biomedical applications. In [19], Youssef et al. proposed a balanced efficiency/gain dual-band antenna design for THz frequencies. An improved sensitivity and directivity-oriented bio-magnetic sensor inspired by the design of metamaterials was proposed by Kumar [20]. Upender and Kumar. [21] studied the incorporation of dielectric materials into FRET systems that exhibit significant enhancements of sensitivity and compatibility for biosensing.

However, in [22], Gonzales et al. present a broad overview of glucose-monitoring technologies from invasive to minimally invasive and non-invasive methods. Through their work, they emphasize that innovative sensor designs for FRET-based systems, when resonated by emerging technologies like THz spectroscopy, will reinforce and boost the accuracy of diagnosis in patients. Although the references above focus on the THz band, the current work transitions this concept to the optical frequency domain (~526 THz), corresponding to the FRET emission spectrum, in order to achieve compatibility with visible-light biosensing mechanisms.

Building on the advancements discussed in the earlier section, the glucose monitoring field continues to evolve with the transition from traditional invasive methods to advanced non-invasive approaches. In the last 10 years, recent devices [23] (e.g., transdermal patches, transcutaneous glucose sensors, and glucose-sensing breath analyzers) providing continuous and painless glucose monitoring have been developed as noninvasive alternatives to traditional glucose monitors that require direct blood contact. Instead, these innovations have used indirect biological measurements, helping to form an easier and more comfortable point of access.

Due to the special distance-dependent energy transfer mechanism, fluorescence resonance energy transfer (FRET) is a fundamental basis for biosensor development. The ratio of FRET (Fluorescence Resonance Energy Transfer) sensors pairs a donor and acceptor molecule for glucose, and the concentration of glucose is detected due to changes in the efficiency of energy transfer between donors and acceptors, enabling high sensitivity and non-invasiveness [24]. This approach has paved the way for the combination of FRET-based biosensors with sophisticated biomedical systems.

In addition to THz-based antenna studies, recent advancements have explored the use of optical antennas in biosensing applications. Foundational works such as Novotny and van Hulst [25] and Gwo et al. [26] demonstrated the potential of light antennas and plasmonic nanostructures for nanoscale signal detection. Applications in biosensing platforms have been expanded in more recent studies that reaffirm their relevance. In [27], Koseoglu et al. gave out biosensors based on plasmonic nanostructures to detect biological sensing in terms of wavelength, angle, phase, and polarization optically. Liu et al. [28] used a plasmonic effect-based design of optical nanogap antennas for miRNA biomarker detection. A visible range plasmonic interaction-enhanced metasensor that provides Fano resonance cooling glucose detection was co-developed with the authors of [28]. The results from these studies validate that visible frequency antenna systems, including the one developed in this work, can be a practical and useful option for compact and sensitive biosensing.

Photodetectors are an important component of biosensing systems, but they are limited by the challenges associated with use in confined environments such as endoscopic capsules. These difficulties are miniaturization, environmental stability, and high spectral sensitivity in ranges suitable for FRET applications. Nevertheless, such optimized photodetectors are realized by advanced CMOS and SiPM with superior sensitivity and much higher reliability and performance stability under such circumstances [29,30,31].

Similarly, major developments in non-invasive glucose monitoring have also been reported in optical and EM-based sensors. Such methods include multi-wavelength light sources such as those used for near-infrared spectroscopy, where blood glucose concentrations can be determined without direct contact with blood, as explained in [32,33]. At the same time, easier-to-use sensors (electromagnetic sensors) are being developed for 24 h monitoring of glucose, which is essential for an artificial pancreas system [34].

Hydrogel and FRET-based biosensors specifically respond to glucose levels and are known to be very reliable [35,36]. Moreover, studies on radiation’s impact on photodetectors, especially CMOS technologies, demonstrate that sensor designs must be optimized to suit integration into medical systems [37,38]. Biosensors are also being used to develop advanced technology solutions such as ingestible wireless devices, illustrated by a modular pH sensor for gastrointestinal monitoring [39,40].

These are just a few examples from this burgeoning field that underscore the promise of merging advanced biosensors with wireless and modular technologies. These developments have changed the landscape of the field, making sure future devices are able to be accurate, reliable, and comfortable for patients.

## 3. Methodology

The methodology section describes the simulations used to optimize photodetectors for endoscopic capsule FRET-based glucose biosensing.

**Figure 1:**
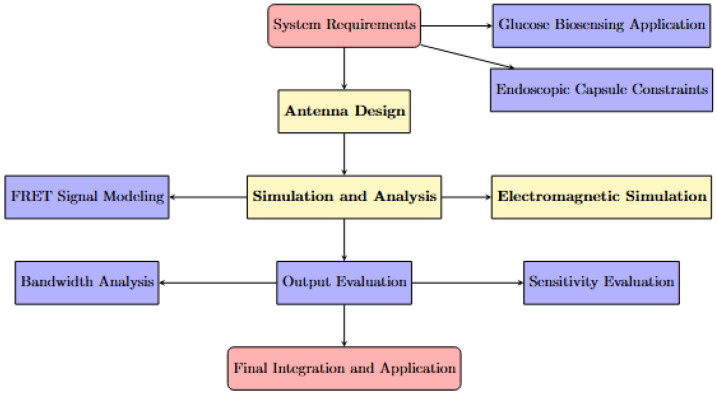
Overview of the methodology for the development of a FRET-based biosensor system.

FRET-based glucose biosensor with the optimized optical frequency antenna Figure 1. The System Requirements, which are the glucose biosensing applications and endoscopic capsule constraints, have been presented in our previous work [41]. This research work mainly deals with Antenna Design and Simulation and Analysis, which are discussed subsequently with an emphasis on electromagnetic Simulation and optimization to match the FRET rules. The Output Evaluation and integration to glucose monitoring systems will be directed by the results.

CST Studio Suite was chosen for its excellent electromagnetic simulation characteristics, which are essential for portraying photo detectors’ designs. The section lists the original design’s entitlements and deviations based on the initial simulation findings, as well as iterative antenna configuration refinement methods. Simulation setup, methodologies, and data pinpointing at different phases are also defined. This method ensures that simulations follow the literature and fulfill actual application requirements.

### 3.1. FRET Efficiency and Frequency Determination for Glucose Biosensing

Förster Resonance Energy Transfer (FRET), which is a distance-dependent mechanism associated with the energy transfer between two light-sensitive molecules namely donor and acceptor, plays an important role in glucose biosensing in combination with the concept of competitive binding theory. FITC (Fluorescein Isothiocyanate) serves as the donor and TRITC (Tetramethylrhodamine Isothiocyanate) acts as acceptor in this application. FRET takes place when non-radiative dipole–dipole transfers energy from donor to the acceptor which results in fluorescence predominately in one wavelength, depending on the distance between two reacting species [22,42].

#### 3.1.1. FRET Calculation

FRET is then defined by the efficiency of FRET, which is expressed as a ratio of energy transferred from the donor to acceptor (Equation (1)).

(1) $$E=\frac{R_{0}^{6}}{R_{0}^{6}+r^{6}}$$

where

- E is the FRET efficiency.
- $R_{0}$ is the Förster radius, the distance at which energy transfer efficiency is 50%.
- r is the distance between the donor (FITC) and acceptor (TRITC).

The Förster radius ($R_{0}$) is dependent on the spectroscopic overlap between the donor emission spectra and the acceptor absorption spectra and can be defined mathematically by Equation (2).

(2) $$R_{0}=0.211\times {\left(\kappa^{2}n^{-4}Q_{D}J\right)}^{1/6}$$

where

- $\kappa^{2}$ is the dipole orientation factor (typically $\kappa^{2}=2/3$ for random orientations).
- n is the refractive index of the medium.
- $Q_{D}$ is the quantum yield of the donor.
- J is the spectral overlap integral, which quantifies the overlap between the emission spectrum of the donor and the absorption spectrum of the acceptor.

Due to the relatively small Förster radius of FRET in aqueous solutions (approx. 5 to 6 nm for FITC and TRITC), FRET is a molecular distance-based method that renders it sensitive enough to establish accurate glucose concentrations at nanometers apart positions. Varying glucose level affects the donor–acceptor distance via competitive bidding and therefore has a direct impact on energy transfer efficiency.

#### 3.1.2. Frequency Specification Based on FRET Efficiency

In this context, the operating frequency for the antenna system is given by the acceptor emission peak (TRITC) following energy transfer. In FITC and TRIC, the emission spectra peaks are ~520 nm (for FITC) and 570 nm (TRIC). The frequency (f) and energy of the emitted photons are related by Equation (3) [43,44,45,46].

(3) $$f=\frac{c}{\lambda }$$

where:

- f is the frequency.
- c is the speed of light $\left(3\times 10^{8}\;m/s\right)$.
- λ is the wavelength of the emitted light (in meters).

For TRITC (emission at 570 nm):

(4) $$f=\frac{3\times 10^{8}\;m/s}{570\times 10^{-9}\;m}\approx 5.26\times 10^{14}\;Hz\;(526THz)$$

This frequency is within the range (operating frequency for antenna system developed to FRET-based glucose biosensing).

In FRET detection of glucose, the donor (FITC) and acceptor (TRITC) are conjugated to a glucose-binding protein or other equivalent structure. With the presence of glucose, competitive binding takes place and replaces donor or acceptor, which influences the distance between donor and acceptor, thus varying the FRET efficiency [8]. With an increase in glucose concentration, the distance between donor and acceptor decreases, which results in higher FRET efficiency. After detection of an apparent shift in fluorescence emission, the FRET signal diminishes as the glucose concentration increases and hence can be quantified. In FRET glucose detection, glucose competes with a glucose-binding protein at higher concentrations to cause separation of the donor (FITC) and acceptor (TRITC), thus resulting in the decreased FRET efficiency. The lower degrees of FRET, which translate into a decreased FRET signal, as identified by a shift in fluorescence emission, enable the quantitative measurement of glucose concentration [41].

The FRET efficiency versus D-glucose concentration relation could be fitted to the following Equation (5) [12].

(5) $$E=E_{max}\left(\frac{\left[G\right]}{K_{d}+[G]}\right)$$

where

- $E_{max}$ is the maximum FRET efficiency.
- [G] is the glucose concentration.
- $K_{d}$ is the dissociation constant of the glucose-binding protein.

By measuring FRET efficiency and fluorescence emission as they relate to antenna operational frequency, real-time detection of glucose concentrations is possible with this model.

These initial estimations from the mathematical FRET efficiency model and distance-proportional relationship of glucose to donors–acceptors allow for accurate mechanisms that address non-invasive glucose sensing in an endoscopic capsule. This approach optimized the antenna design to work from 500 to 550 THz, which is effective for detecting FRET-based fluorescence signals and hence allows reliable glucose measurement in the interior of an endoscopic capsule.

In this system, the antenna does not enhance the FRET process itself, which is a molecular interaction, but rather the antenna serves to detect and/or transmit the information in the form of an optical signal that is generated by FRET. The antenna, specifically designed in order to be resonant to the emission frequency of TRITC (~526 THz), is able to capture the optical signal emitted from the energy transfer process. This alignment improves signal collection or propagation in the environment of the endoscopic capsule, resulting in the observation of real-time monitoring of FRET changes after glucose administration. Thus, the antenna serves as an interface that translates molecular-scale energy transfer dynamics into macroscale readout data.

### 3.2. Simulation Setup and Tuning Design

The antenna was tuned up for working range from 500 THz to 550 THz. This was achieved by first calculating the antenna’s resonant frequency based on the following Equation (6) for mathematical model [47].

(6) $$f_{r}=\frac{c}{2L_{e}\sqrt{\epsilon_{r}}}$$

where

- $f_{r}$ is the resonant frequency.
- c is the speed of light $\left(3\times 10^{8}\;m/s\right)$.
- $L_{e}$ is the effective length of the patch antenna.
- $\epsilon_{r}$ is the relative permittivity of the substrate.

It is well known that Equation (6), originally intended for microwave patch antenna resonance, is incomplete when considering materials at optical frequencies, as dispersion, plasmonic effects, and non-ideal conductor behavior become more pronounced. The equation was only used in this study as an initial estimation tool guiding the dimensional parameters of the antenna. A full-wave simulation using CST Studio Suite was then conducted to validate and optimize the actual electromagnetic response (i.e., resonance accuracy, impedance matching, and radiation efficiency). Although the formula of the guided-mode resonance formula is essentially classical, this approach ensures that the final design operates as it should in the visible frequency region (~526 THz).

The physical length $L_{p}$ of the patch antenna, combined with the fringing effects at the edges, results in an effective length $L_{e}$, as expressed in Equation (7). The effective length $L_{e}$ of a patch antenna is defined as consisting of the physical length $L_{p}$ of the patch and additional effects of the fringing fields on the edges of the antenna. The fringing effect arises due to the incomplete confinement of the electromagnetic fields within the patch, the field extends outside the physical limits of the patch and most significantly so, at the edge. These impacts combine into the characteristics and function of the antenna, including resonant frequency.

(7) $$L_{e}=L_{p}+2∆L$$

where ∆L is the additional length is due to fringing fields, calculated as Equation (8).

(8) $$∆L=0.412h\left(\frac{\epsilon_{r}+0.3}{\epsilon_{r}-0.258}\right)\left(\frac{W_{p}/h+0.264}{W_{p}/h+0.8}\right)$$

Here, h is the thickness of the substrate, and $W_{p}$ is the width of the patch antenna. The width $W_{p}$ of the patch is calculated as

(9) $$W_{p}=\frac{c}{2f_{r}\sqrt{\frac{\epsilon_{r}+1}{2}}}$$

The process of tuning the antenna includes adjusting its dimensions (like patch width and length) to achieve better performance. This process, at the nanoscale, is more sensitive than millimeter (mm) scale designs. However, the difference between millimeter-scale size and nanometer-scale design is that at nanometer scale there can be significant impact on performance parameters like resonant frequency, impedance matching, and radiation efficiency to even a small dimensional change.

For instance, tuning by 0.1 mm in patch length or width provides minimal shifts in frequency or impedance in millimeter-wave designs, but induces huge performance change in nanoscale designs when similar adjustments are made at only a few nanometers, making it a more delicate process. The reason for this difference can be primarily attributed to increased operational frequencies and accompanying shorter wavelengths in nanoscale applications.

Dimensions in Table 1 were obtained through an iterative procedure of performing simulations via CST Studio Suite and theoretical calculations. To achieve proper radiation from the patch antenna at target frequencies, optimum dimensions of width and length were simulated. The values of impedance matching and VSWR were also optimized to be less than 2 in order to minimize the reflection coefficient to enable maximum power transfer. The resonant frequency (f_r_) was expressed as a function of the physical dimensions of the antenna, where patch width (W_p_) and length (L_p_) were calculated using the equations mentioned above with other important parameters such as feed width and substrate thickness for optimal performance. A copper patch and a quartz substrate were selected because of their good conductivity for the metal and good dielectric properties in the visible optical frequency range. Copper has been shown to have greater Ohmic losses in the optical regime compared to noble metals like gold, but our initial simulations showed similar resonance behavior and gain between the two metals. Due to the ease of convergence in the modeling, the compatibility of CST’s modeling parameters with copper, and since many copper models exist for optical frequency simulations, copper was used to simplify calculations and allow for quicker simulations. However, the optical loss property is well-respected, so we may introduce other materials in later experiments.

**Figure 2:**
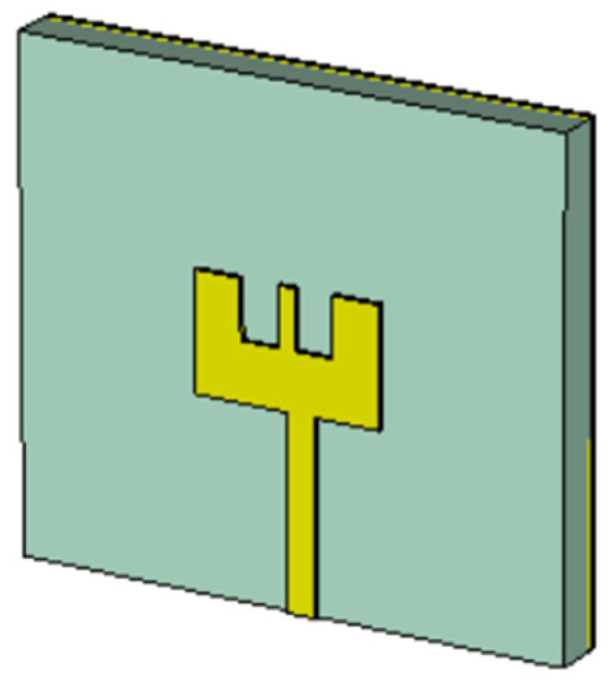
Basing antenna design for FRET resonance frequency.

As illustrated in Figure 2, which shows the original design as can be seen from the results obtained, Satisfactory performance of less than −16 dB is recorded for the S11, and the Voltage Standing Wave Ratio (VSWR), which was lower than 2, indicates proper impedance matching. The gain was conditioned to be higher than 4.2 dBi, which is suitable for the preliminary simulations undertaken.

**Figure 3:**
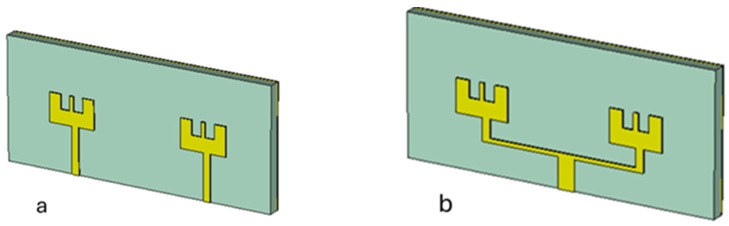
Array designs: (**a**) description of the array for the first design; (**b**) description of the array for the second design.

Nevertheless, in order to improve the behavior—specifically to increase gain and directivity (both important for effective detection of the FRET signal when it comes to glucose biosensing applications)—an array design was proposed (Figure 3). The matrix setup enabled the coping of dense photo detection elements required to maximize signal transference in the confined space of an endoscope capsule.

Hence, these antenna designs can drop in integration by simply increasing the number of elements in the array to achieve high directivity and gain ensuring coverage and strength vital for biomedical applications on FRET-based biosensing for accurate glucose level detection.

### 3.3. Design Process

For the array design phase, two variants of the configuration were simulated. The purpose was to evaluate each’s performance enhancements with the original single patch configuration. The results are shown in Figure 3, with different geometrical alignments and spacings of the array configurations. Each design was developed to perform better in terms of the directional gain and overall efficiency, which were integral for increased accuracy of detection using relatively simple photon emission arrays, given endoscopic capsules’ compact operation space.

First, it is important to explore the simulation parameters for both configurations. These Figs were carefully selected based on the frequency range of operation and other specifics of the FRET-based biosensing process.

**Figure 4:**
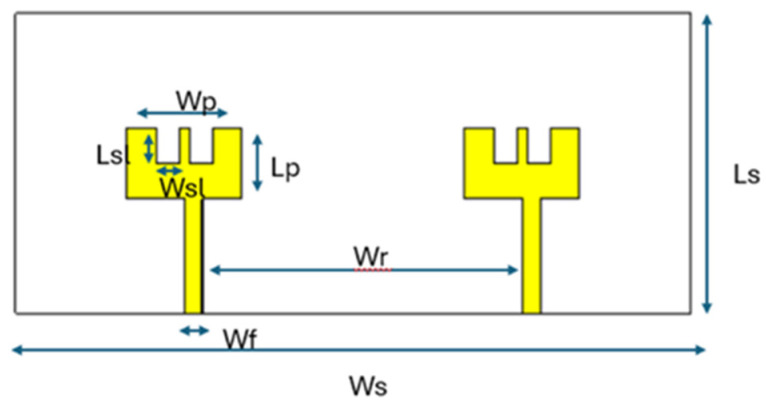
Array 1 detailed design.

**Figure 5:**
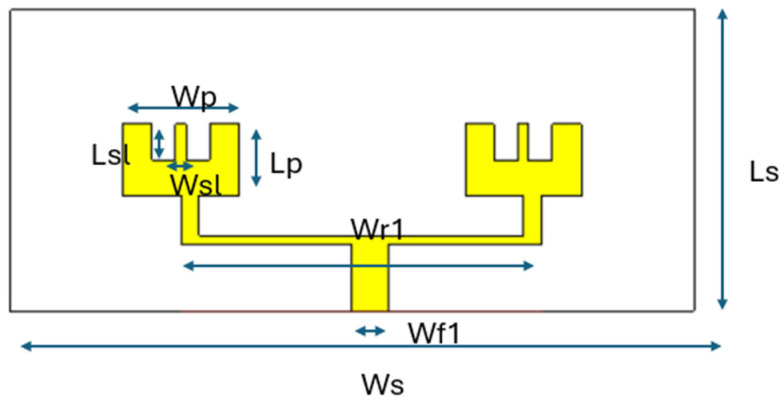
Array 2 detailed design.

Table 1 provides a summary of key dimensions for designs created in Figure 4 and Figure 5, including W_p_, L_p_, W_s_, L_s_, and other important variables. The simulations’ success critically depended on correctly defining these properties, which determine the resonant frequency, impedance matching, and radiating power of each antenna array. The configuration was implemented in CST Studio Suite for each. Following this step, the behavior of each setup under selected operational conditions was studied with regard to S-parameters, VSWR, and gain. Data for antenna scanning were provided from configurations. The specific aim of the results was to establish which of the simulations would display the best performance in terms of beam steering and gain enhancement, factors important for peripheral medical application.

### 3.4. Simulation Parameters and Execution: Array Design

**Figure 6:**
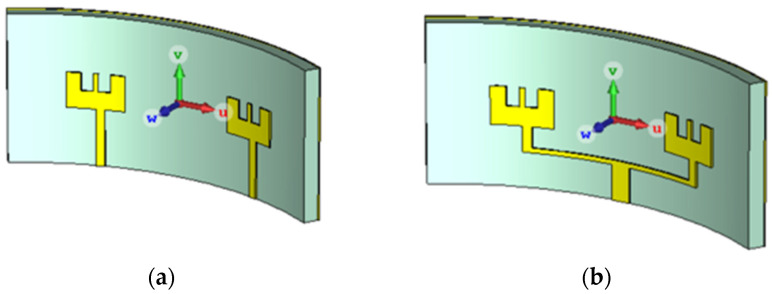
Array designs after binding: (**a**) description of the array for the first design after binding; (**b**) description of the array for the second design after binding.

One of the important parts was the integration and bending of the antenna designs. These steps were critical in simulating real-world application scenarios. As such, the antenna would have to be integrated to accommodate both limited and curved internal spaces. Figure 6 (part a) demonstrates the bending of the first antenna design, and Figure 6 (part b) shows the bending of the second one. More specifically, both designs have been bent around the cylinder with a 1000 nm radius. The main reason for simulating this process has been the necessity to establish the impact of mechanical stress and curvature on the antenna’s performance. The result would allow a deeper understanding to be gained of whether an antenna can be durable and functional when deployed in a biological environment that can be considered dynamic.

It is vital to add that the current simulations have been conducted to realize how the bending of a flat antenna influences the radiation pattern, impedance, and resonant frequency. Such parameters have been monitored due to their significance for maintaining the ability of the antenna to communicate and sense when placed in the endoscopic capsule. As such, each antenna design has been carefully portrayed in CST Studio Suite, and both have been devised to bend around the specified form. Then, a shift in the performance of the design has been monitored to realize how differential forms affect it. It can now be confirmed that the results would offer an accurate picture of how both designs are both mechanically and electromagnetically sturdy, and each could be implemented to the fully desired outcome.

## 4. Findings

This section evaluates the results of comprehensive simulations comparing two antenna array designs in their original and bent versions. These results focus on S-parameters, gain, power distribution, and Voltage Standing Wave Ratio, which determine whether each antenna is suitable for FRET-based glucose sensing with FITC and TRITC in an endoscopic capsule environment. Therefore, an advanced comparative analysis of the acquired data can determine the best antenna design to meet the most challenging environmental specifications and identify specific practical implications regarding antenna configuration and robustness for the development of non-invasive biosensing technologies.

### 4.1. Results

**Figure 7:**
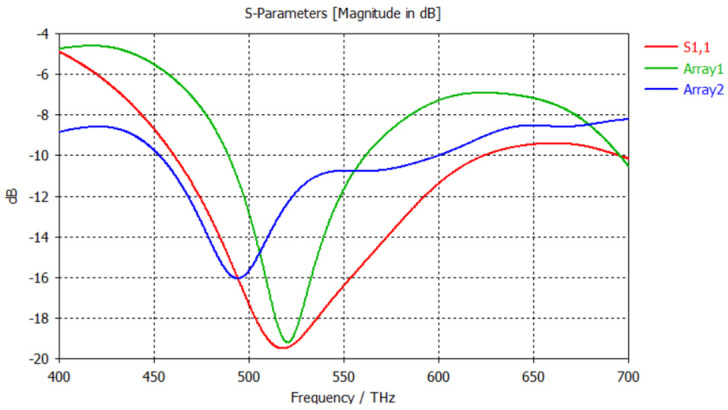
S11 results for array design antennas.

**Figure 8:**
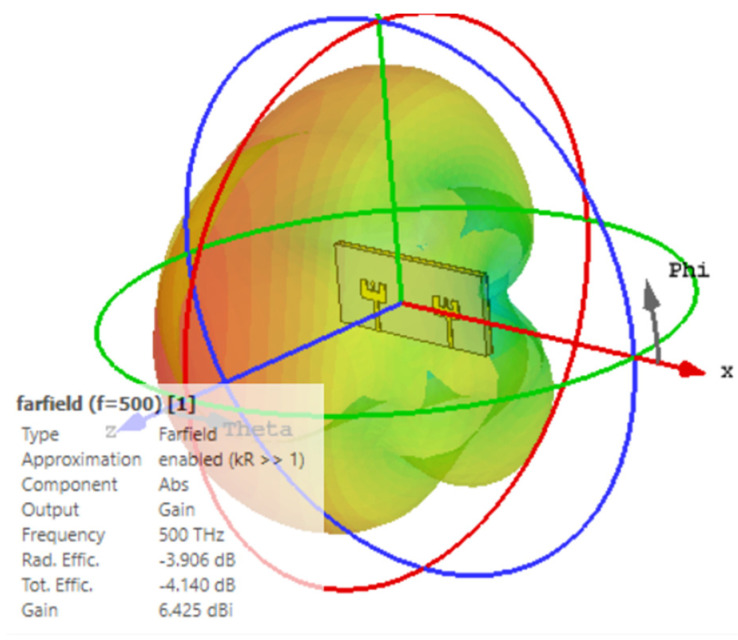
Array 1 far-field 500 Thz results.

**Figure 9:**
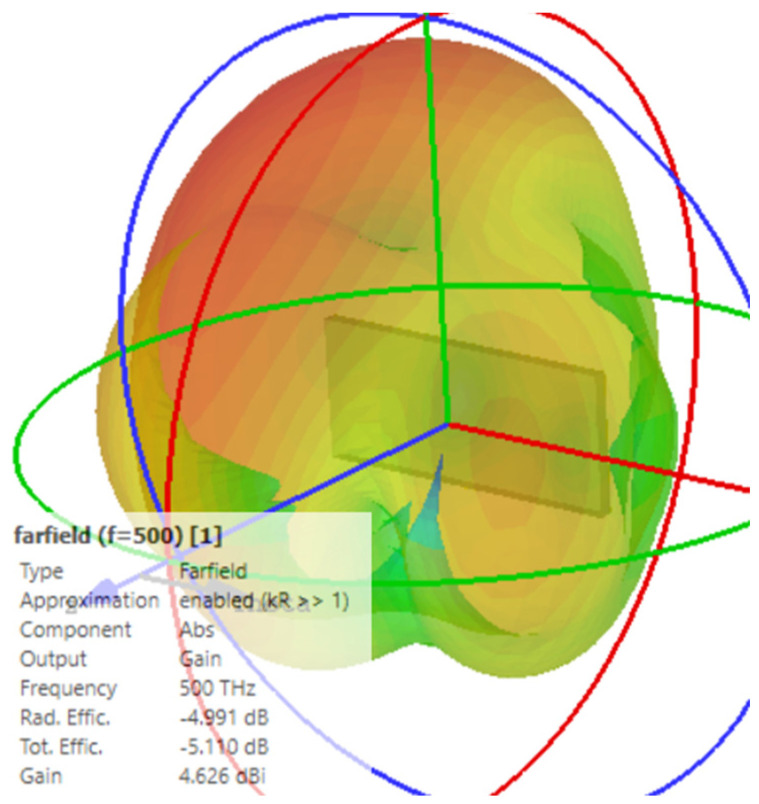
Array 2 far-field 500 Thz results.

**Figure 10:**
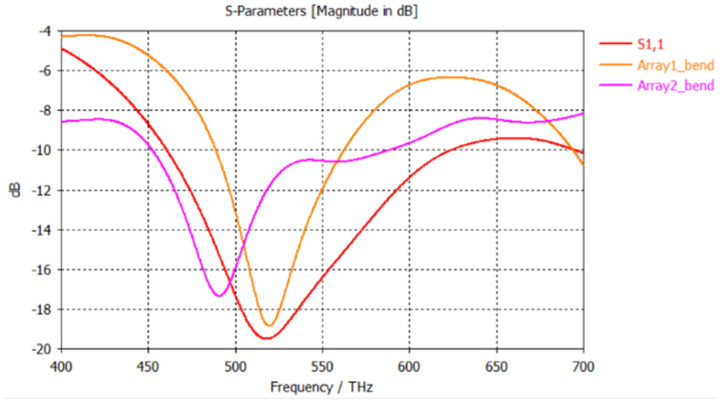
S11 results for bent array design antennas.

**Figure 11:**
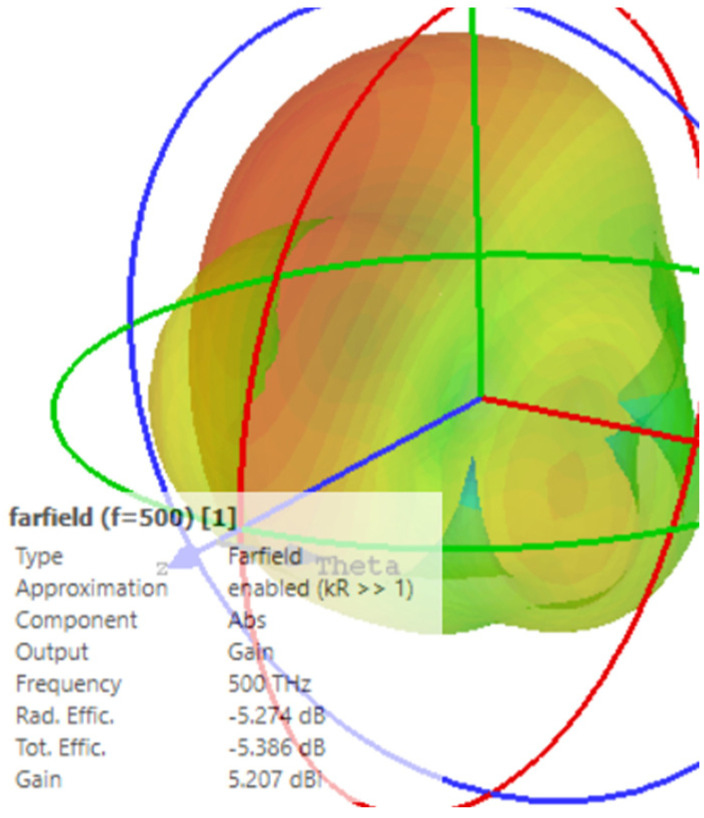
Bended version of Array 1 far-field 500 Thz results.

**Figure 12:**
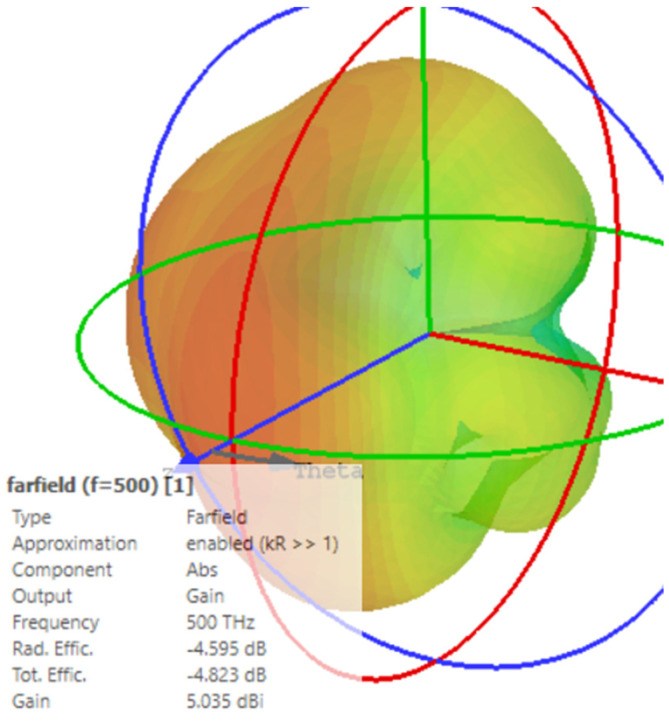
Bended version of Array 2 far-field 500 Thz results.

**Figure 13:**
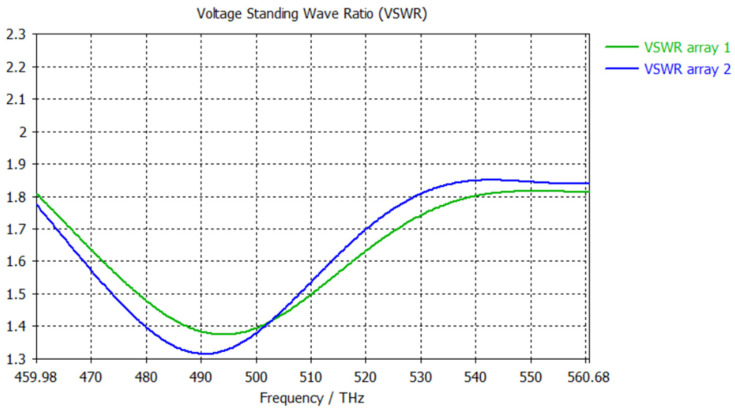
Bended version of Arrays 1 and 2 VSWR results.

The result of the simulation, which is shown in Figure 7, Figure 8, Figure 9, Figure 10, Figure 11, Figure 12 and Figure 13, is a thorough check of the realized antenna’s characteristics before and after the bending. As seen in the S-parameters for Array 1 and Array 2 before bending, featured in Figure 7, Array 1 demonstrates a more stable performance over the target frequency band, which for this case is 500–550 THz. This statement is related to obtaining −16 dB for S11 of Array 1, which demonstrates that the antenna is effective and usable for the target application.

The modified S-parameters for both versions, while analyzing the bending of the antennas, are shown in Figure 10. Both designs show a modification in their S-parameters but continue to maintain an effective performance level with minimal degradation, indicative of their robust design for practical deployment scenarios.

As seen in Figure 11 and Figure 12, both antennas demonstrate reduced gains after bending, reaching 5.2 dBi and 5.035 dBi, compared to the pre-bending characteristics of 6.425 dBi and 4.62 dBi for Array 1 and Array 2, respectively.

In addition, with the VSWR results, as shown in Figure 13, being less than 2.2 after the bending, my antennas match the requirements for impedance well enough to be accepted as effective radiating systems.

The analysis of the results substantially supports Array 1 for addressing the needs of the specified application, which is FRET-based glucose sensing with the use of FITC and TRITC. In particular, the findings depicted show that the sensitivity and specificity of the antenna’s operation is significantly higher for the proposed solution. One should note that the results presented in the context of S-parameters seem to remain to be more stable when working with Array 1, which is also related to the higher gain of this particular option. Although both units are characterized by effective performance, especially in terms of the VSWR level and power management, it is suggested that the advantages of the first design should be considered a stronger argument for its selection.

Overall, it is possible to ascertain that both antennas are appropriate for the target application, but Array 1 is characterized by some features correlated with a more effective addressing of the problem. In particular, this design is more successful in terms of exercising higher power capacity, lower VSWR, and greater gains regardless of the identified physical structure change. Overall, it can be viewed as a perfect opportunity for improving the performance of FRET-based biosensing and its ability to provide accurate data on changes in glucose concentration.

### 4.2. SAR Calculation and Sensing Capabilities

**Figure 14:**
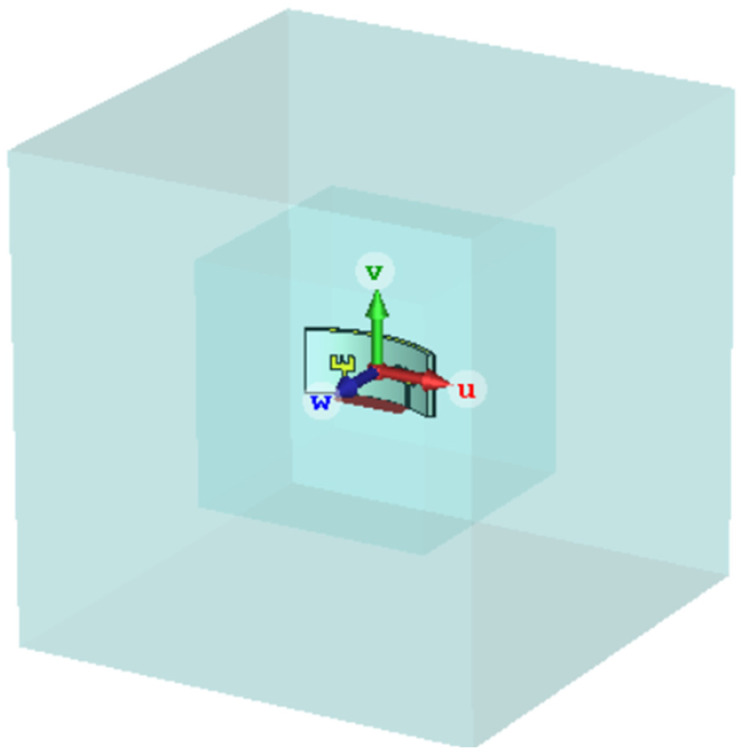
Antenna in the medium.

The antenna was tested in a virtual measurement setup surrounded by three different media: (i) water; (ii) low-glucose medium; and (iii) high-glucose medium, shown in Figure 14. For the proposed work, water medium was selected because of its dielectric characteristics with a density of 1000 kg/m^3^ and relative permittivity ~80 [45]. The density of the low-glucose medium (1100 kg/m^3^) modeled an intermediate glucose level, and that of the high-glucose medium (1200 kg/m^3^) mimicked a glucose-rich environment with a higher corresponding dielectric constant [46]. These media were specified and parameterized to represent realistic bio-conditions for FRET-based glucose sensing scenarios [48]. Table 2 provides the detailed properties of each medium, such as density and dielectric constants, so it can be guided to represent its particulars in simulation [49,50].

**Figure 15:**
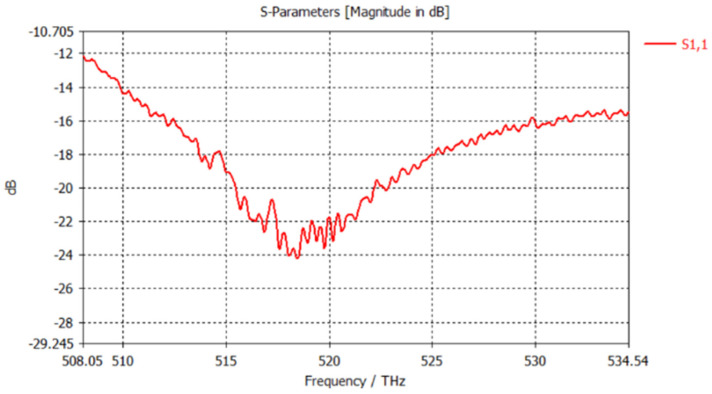
Antenna in the medium S11 results.

**Figure 16:**
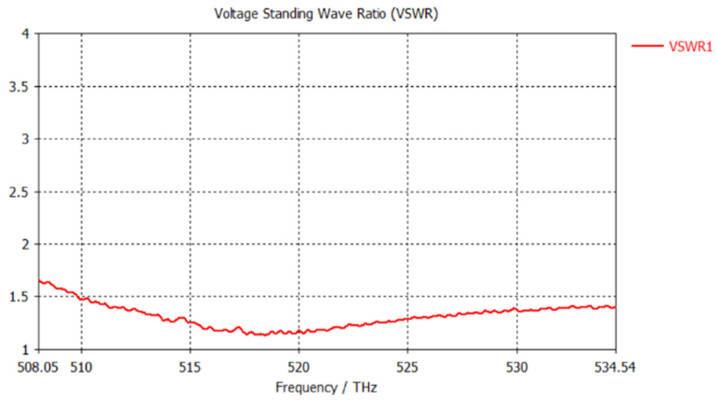
Antenna in the medium VSWR results.

A few tweaks of mesh tuning later, and the simulation showed negligible changes to return loss or VSWR, supporting that little change in expressed performance was exhibited within any combination of media. S11 parameter and VSWR performance shown in Figure 15 and Figure 16, which always have consistent behavior with return loss remaining less than −15 dB, confirm good impedance matching. In this medium, the gain of the antenna was also affected and attained up to 9 dBi (See Figure 16), indicating an improved far-field pattern from which it can be concluded that glucose in the medium resulted in better radiation efficiency of the antenna [51,52].

**Chart 1:**
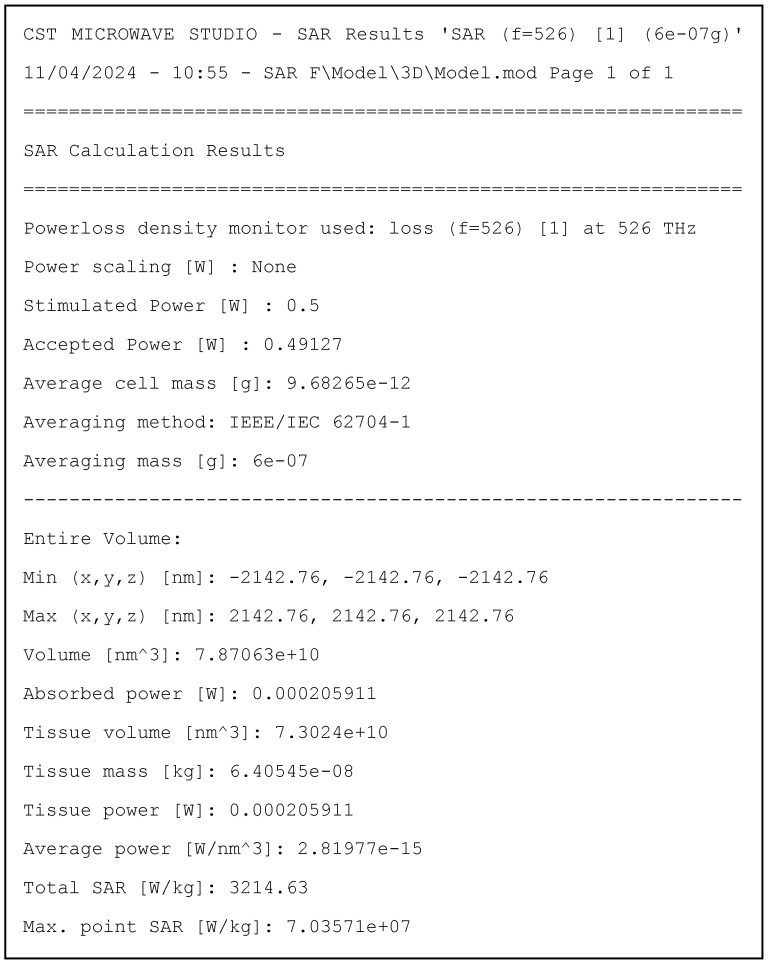
CST results.

The nature of large media made SAR (Specific Absorption Rate) analysis difficult because the computation required to simulate was extensive, and CST Studio Suite imposes limitations on the size (1 × 10^−7^ g < averaging mass < 5000 g) [53], which needed to be appropriately configured and optimized for accurate analysis. However, the simulation results were a success despite these challenges (Chart 1, Textbox). The peak SAR value of ~1.92 W/g at (−51.9895, −452.916, 366.667), which coincides with high-glucose region specs, also reflects effective energy absorption in regions that replicate the glucose-rich microenvironment. The analysis further confirmed that the antenna operates properly, wherein there is proper distribution of energy absorption within a certain region, confirming safe operation of the antenna (Chart 1). These results support the antenna’s ability to operate in environments saturated with glucose and validate its potential as a FRET-based biosensing application.

## 5. Conclusions

A carefully designed, structurally flexible antenna design enables a FRET (Förster resonance energy transfer)-based biosensor system within endoscopic capsules with the potential of non-invasive glucose monitoring. The antenna simulated embedded in a glucose-rich layered medium can help design low-cost, reliable antennas that provide stable impedance matching and high gain needed for significant glucose detection. The safe operational limits for energy absorption are confirmed by the SAR calculations, reiterating the safety and reliability of the design. Its mechanical tunability and uniform functionality at high glucose concentration demonstrate its promising applications for biosensing via highly stable FRET and could provide new insight for non-invasive and real-time detection in biomedical sciences. This model has the potential to be utilized for clinical use after further experimental validation. This is a proof of concept based on simulation. Although this study did not implement direct physical sensitivity measurements, further work will be directed towards constructing the proposed design and experimentally validating its performance under realistic biosensing conditions.

It is important to clarify that, due to the limited penetration depth of visible light at ~526 THz (typically less than 1 mm in biological tissue), the proposed system does not target blood glucose directly. Rather is for measuring glucose in interstitial (intercellular) fluid, where it strongly correlates with blood glucose and is optically accessible. This clarification aligns the proposed model with the physical constraints of light–tissue interaction and reflects the approach used in several existing non-invasive glucose monitoring systems.

## Figures and Tables

**Table 1:** Dimensions of the final design.

Notation	Dimension in nm	Notation	Dimension in nm
Ws	1146	Ls	1012
Wp	194.5	Lp	120
Wf	30	Wf1	80
Wsl	40	Lsl	60
Wr	542.96	Wr1	602.96

**Table 2:** Medium settings.

Medium	Density (kg/m^3^)	Relative Permittivity (εr)	Conductivity (S/m)	Tangent Delta
Water	1000	80	0.001	0.15
Low Glucose Medium	1100	76	0.002	0.15
High Glucose Medium	1200	77	0.003	0.15

## Data Availability

All data supporting the findings of this study, including simulation results, design parameters, and analysis figures, are included within the manuscript.
